# Intractable nausea and vomiting in naïve ingestion of kratom for analgesia

**DOI:** 10.1186/s12245-020-00301-0

**Published:** 2020-08-05

**Authors:** Vir Singh, Nadir Mulla, James Link Wilson, Aaron Umansky, Jenny Lee, Trilok Stead, Latha Ganti

**Affiliations:** 1 University of Central Florida College of Medicine, Ocala Regional Medical Center, Ocala, FL USA; 2 Envision Physician Services, Clearwater, FL USA; 3 University of Texas Medical Branch at Galveston, Galveston, TX USA; 4 Ocala Regional Medical Center, Ocala, FL USA; 5 Brown University, Providence, RI USA; 6 Trinity Preparatory School, Winter Park, FL USA

**Keywords:** Kratom, Opioid epidemic, Opioids

## Abstract

**Background:**

Kratom is a habit-forming opioid-like substance with an acute toxidrome of various symptoms such as diaphoresis, dizziness, nausea, and vomiting. Chronic users require increasing dosages for the analgesic effects. Although kratom use dates back to the 1800s in Asia, kratom intoxication is still a novel (but increasing) toxidrome in the Western world. Here, we present a novel case of acute toxicity from overdose in a kratom-naïve patient, taking place through recommendation by a family member who chronically takes this substance.

**Case presentation:**

We present the case of a 62-year-old woman arriving to the emergency department (ED) with a chief complaint of intractable vomiting after ingestion of kratom. After a day of yard work, she was in pain, secondary to her osteoporotic joints. She was recommended kratom from a family member, who stated he was using kratom to transition away from opioid dependence. She took two “scoops.” She proceeded to have multiple episodes of vomiting at home. She came to the ED, where she required multiple rounds of anti-emetic medication for resolution of her symptoms.

**Discussion:**

We present a classic case of a novel acute toxicity: kratom. A unique aspect of this case is the circumstance by which this toxicity took place: a family member who chronically takes this substance (that requires increasing dosages to remain effective) recommended a dosage to this kratom-naïve patient, leading to overdose. This opioid family alternative substance is gaining popularity across the USA in the era of the opioid crisis. Further documentation of case reports and research is required to learn the associated risks of the use of this substance.

## Background

**Fig. 1:**
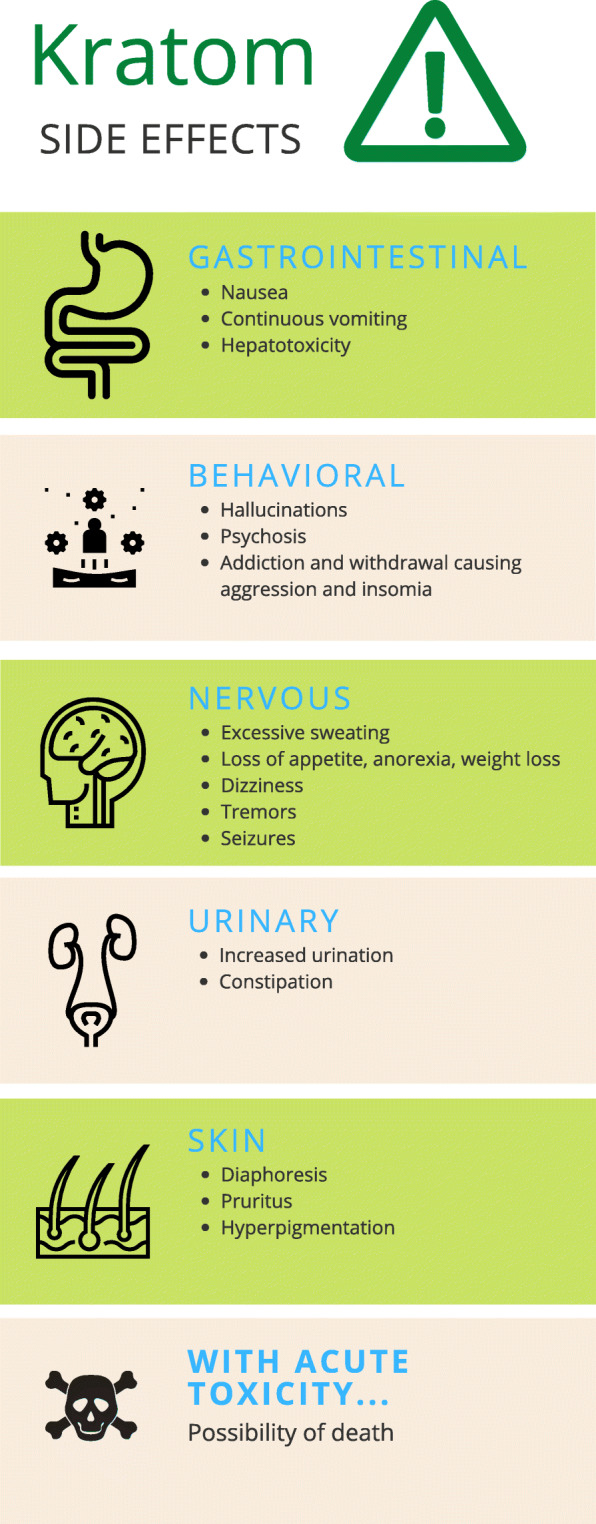
Kratom side effects

Kratom is a tree native to Southeast Asia known for its psychotropic properties and is easily obtained over the Internet. Kratom can be ingested in many ways, including being taken as a pill, an extract, being smoked, put in food, or even sold as a gum. It is sometimes sold as a green powder in packets labeled “not for human consumption.” It has many street names, such as biak, ketum, kakuam, ithang, and thom [1]. There have been many deaths attributed to kratom, though most of the victims had also taken other substances that may have caused death [2]. Kratom can be classified as both an opioid and a stimulant. Two compounds that are found in kratom, mitragynine and 7-a-hydroxymitragynine, interact with opioid receptors in the brain, which can produce sedation, decreased pain, and pleasure, usually when large amounts are consumed. However, when small amounts are consumed, users reported increased energy, sociability, and alertness. Kratom’s unpleasant effects (Fig. 1) include nausea, itching, sweating, dry mouth, constipation, increased urination, loss of appetite, and hallucinations [1].

Kratom has been used in Southeast Asia for years and overdose deaths are not nearly as present as they are in the USA, although tolerance and dependence have been reported in the region. Kratom-related deaths in the USA almost always involved other factors, such as a pre-existing condition or, as previously mentioned, another drug being taken with the kratom. Last year, the FDA found salmonella in shipments of kratom to the USA, resulting in over 50 hospitalizations. In a fatal car crash case, the autopsy revealed that the victim was under the influence of elevated levels of caffeine and mitragynine [2]. Here, the authors discuss the case of intractable vomiting and nausea caused by kratom intoxication incorporating the CARE guidelines [3] and the International statement recommending against the use of terminology that can stigmatize people [4].

## Case presentation

The patient is a 62-year-old woman with a history of chronic obstructive pulmonary disease and asthma who presented to the emergency department (ED) via emergency medical service (EMS) with a chief complaint of vomiting, nausea, and abdominal cramping that started 4 h prior to her arrival. The patient stated that she had spent the day exerting herself performing various outdoor gardening and yard maintenance activities. She did not remain hydrated during this time. Additionally, she felt progressive musculoskeletal lower extremity joint pain as she mowed the lawn. She consulted her family for modalities of pain relief. Her son in law recommended kratom, which he was allegedly prescribed by a pain management practitioner who is attempting to transition him from opioid dependence to an alternative substance. The patient took two “scoops” (each estimated by the patient to be approximately a teaspoon) of a commercially available powdered preparation. She had never consumed kratom previously. At 11:30 pm, she felt nauseated, went to bed, and vomited four times—her emesis was non-bloody and non-bilious, consisting of food. She additionally had abdominal pain, which was described as mild upper and lower crampy non-radiating abdominal pain. There was no associated diarrhea or constipation. There was no associated fever. The patient denied other symptoms. The patient’s family noted all other family members ate the same recent meals as the patient, with no family members reporting similar symptoms. Prior to arrival to the ED at 4:27 am, she continued to have nausea and abdominal pain, for which she called EMS and presented to the ED. En route, EMS noted the patient’s vital signs to be stable and a finger stick glucose reading within normal limits.

On arrival to the hospital, her presenting vital signs were a heart rate of 92, respiratory rate of 16, oxygen saturation of 97% on room air, and a temperature of 36.1 °C. On physical examination, she was found to be well appearing with moist mucous membranes. She had tachycardia. She had a soft abdomen with upper abdominal tenderness. Her skin was warm and dry with brisk capillary refill and no rash. A differential diagnosis was entertained that included small bowel obstruction, diabetic ketoacidosis/hyperosmolar hyperglycemic state (DKA/HHS), acute gastroenteritis, food poisoning, pancreatitis, acute cholecystitis and other gallbladder pathology, intracranial pathology, kratom toxicity, and pregnancy/hyperemesis gravidarum. Small bowel obstruction was deemed less likely due to the lack of prior surgeries and normal bowel movements. Acute gastroenteritis was deemed less likely with normal bowel movements. DKA/HHS was deemed sufficiently evaluated for with no history of diabetes and normal EMS blood glucose reading. Intracranial pathology was considered due to vomiting without diarrhea, but was less likely due to associated abdominal pain.

A complete blood count (CBC), basic metabolic panel (BMP), liver function tests (LFT), and lipase were ordered. All CBC values were within normal limits except for a white blood cell count of 11,600 cells/mm^3^. BMP was within normal limits except for elevated blood urea nitrogen (BUN) of 22 mg/dl and glucose of 165 mg/dl. LFTs and lipase were also within normal limits. The leukocytosis was considered to be nonspecific leukocytosis in the setting of vomiting. The BUN was evidence of mild dehydration secondary to vomiting. During her stay, she was medicated with ondansetron 4 mg IV administered at 04:24 and at 04:47, and for refractory nausea, promethazine 12.5 mg IV at 07:00. She was also given famotidine 20 mg IV at 04:46 and 2 l of normal saline during ED stay. She was reassessed at 07:00 and 08:30 and found to have cessation of her symptoms. She was discharged with kratom avoidance precautions as obtained from Internet sources.

## Discussion

“Kratom” is a common term for derivatives of *Mitragyna speciosa*, a tree native to Southeast Asia. Leaves of the tree are prepared in various ways for consumption: they can be chewed, ground into an extract, dried and crushed into a powder, or consumed as tea [5]. Documentation of its use in its native distribution date to the 1830s in Malaysia as an opium substitute [6]. There are many uses for which indigenous cultures utilized kratom, including as a panacea for GI infections and diarrhea, and for boosting energy reserves needed to accomplish manual labor. The main active molecules in kratom are the alkaloids (plant-derived nitrogenous molecules) mitragynine and 7-hydroxymitragynine [7]. Other alkaloids comprising 5–10% of alkaloid content are paynantheine, speciociliatine, and speciogynine. These compounds have mu-, kappa-, and delta-opioid receptor agonism. Kratom appears to have different effects based on dosing. At a lower dose (1 to 5 g), it has been described to have cocaine-like stimulant effects, and at higher doses (5 to 15 g), sedative opioid-like effects. At higher doses, a toxidrome may occur, with diaphoresis, dizziness, and nausea, which gives way to euphoria. Kratom has been reported to be tolerance- and addiction-forming in numerous case reports [5, 7, 8], further evidenced by withdrawal symptoms with attempted cessation. There are adverse effects associated both with chronic use and with acute overdose/toxicity. Chronic users may suffer tremors, anorexia, frequent urination, weight loss, seizures, or psychosis [5]. They may suffer from withdrawal symptoms ranging from aggression to insomnia. Acute toxicity may result in seizure, hepatotoxicity (intrahepatic cholestasis), and possibly death.

Recently, there has been concern of an emerging epidemic of kratom, as its opioid-like properties have led to a resurgence in use in the context of America’s opioid crisis. Patients with opioid use disorders have turned to kratom as a substitute [9]. Kratom use as reported to poison control centers increased 10-fold between 2010 and 2015 [10]. The drug is currently on the Drugs of Concern list of the DEA. It is illegal in Alabama, Arkansas, Indiana, Rhode Island, Vermont, and Wisconsin. Its legal status is in continuous flux as numerous municipalities and states consider bans, while Vermont considers decriminalization of the substance.

We present a case of a kratom-naïve patient who upon consumption of this supplement developed intractable nausea and vomiting. We hypothesize that as opioid-dependent users continue to turn to kratom as an alternative to opioids, and increase their dosage as they develop tolerance, the prevalence of kratom toxicity, and use in naïve patients may continue to increase. Kratom is an emerging opioid substance not yet scheduled by the DEA that deserves further evaluation for its adverse effects, safety concerns, and shifting legal status.

## Abbreviations

ED: Emergency department
EMS: Emergency medical service
DKA: Diabetic ketoacidosis
HHS: Hyperosmolar hyperglycemic state
CBC: Complete blood count
BMP: Basic metabolic panel
LFT: Liver function tests
BUN: Blood urea nitrogen
